# Evaluation of the safety and efficacy of transarterial sevelamer embolization in a rabbit liver cancer model: A challenge on the size rule for vascular occlusion

**DOI:** 10.3389/fbioe.2022.1058042

**Published:** 2022-12-12

**Authors:** Hong Chen, Chuan-Sheng Xie, Yan-Shu Li, Zhi-Qiang Deng, Yang-Feng Lv, Qiu-Chen Bi, Jian-Jun Tang, Rong-Guang Luo, Qun Tang

**Affiliations:** ^1^ Jiangxi Provincial Key Laboratory of Preventive Medicine, School of Public Health, Nanchang University, Nanchang, China; ^2^ Jiangxi Center of Medical Device Testing, Nanchang, China; ^3^ Department of Oncology, The First People’s Hospital of Fuzhou, Fuzhou, China; ^4^ Institute for Advanced Study, Nanchang University, Nanchang, China; ^5^ Department of Respiratory and Critical Care Medicine, The First Affiliated Hospital of Nanchang University, Nanchang, China; ^6^ Department of Medical Imaging and Interventional Radiology, The First Affiliated Hospital of Nanchang University, Nanchang, China

**Keywords:** transarterial chemoembolization, safety, sevelamer, VX2 carcinoma, toxicity

## Abstract

As the most efficient method to treat hepatocellular carcinoma in the immediate or advanced stage, transarterial chemoembolization (TACE) is coming into the era of microsphere (MP). Drug-eluting beads have shown their huge potential as an embolic agent and drug carrier for chemoembolization, but their sizes are strictly limited to be above 40 μm, which was considered to occlude vessels in a safe mode. microsphere smaller than 40 µm is easy to be washed out and transported to the normal liver lobe or other organs, causing severe adverse events and failed embolization. To determine whether sevelamer ultrafine particle (0.2–0.5 µm) is qualified as a safe and efficient embolic agent, we investigated the safety and therapeutic efficiency of transarterial sevelamer embolization (TASE) in the VX2 rabbit liver cancer model, aiming to challenge the “40 µm” rule on the selection criteria of the MP. In a four-arm study, blank bead (Callisphere, 100–300 µm), luminescent polystyrene microsphere (10, 100 µm), and sevelamer particle were transarterially administered to evaluate the threshold size of the MP size for intrahepatic or extrahepatic permeability. Another four-arm study was designed to clarify the safety and efficiency of preclinical transarterial sevelamer embolizationTASE tests over other techniques. Sham (saline), TASE, C-TACE, and D-TACE (*n* = 6) were compared in terms of serum chemistry, histopathology, and tumor necrosis ratio. In the first trials, the “40 µm” rule was detectable on the VX2 cancer model, but the regulation has no application to the new embolic agent as sevelamer ultrafine particles have not been found to leak out from the VX2 lesions, only found in the embolized vessels. Pathology proves that less viable tumor residue was found 2 weeks after the procedure, evidencing a better therapeutic outcome. No adverse events were found except for a short stress response. These results indicate that sevelamer is a safe and efficient embolic as an alternative to the current MP-based embolization therapy techniques.

## 1 Introduction

Interventional radiology has rapidly progressed for around one hundred years. Rapid development in interventional oncology allows for the chemoembolization of those highly vascular tumors including hepatocellular carcinoma (HCC) (Rosch, Keller, & Kaufman, 2003). Each year more than one million HCC patients are treated with chemoembolization therapy, a specific type of procedure that utilizes different embolic agents to occlude the tumor-feeding artery. Embolic agents are normally natural and synthetic polymers and are delivered by the catheters positioned within the tumor-feeding vessels (Poursaid, Jensen, Huo, & Ghandehari, 2016). Clinician interventionists are taking trials for multifunctional embolic agents such as those loaded with anticancer drugs or radioactive nuclides (Nicolini, Crespi, & Martinetti, 2011; Bannerman & Wan, 2016). But the commercial embolic agents are still far from expectation (Coldwell, Stokes, & Yakes, 1994; Osuga et al., 2012), as an ideal agent should be biocompatible, cost-effective, and easily deployed, and rapidly occlude an artery, maximally fill the targeted vasculature. Additionally, recanalization, fragmentation, and dislodgement should be avoided, Moreover, it is capable of carrying anticancer drugs, being visible by X-ray. Finally, it should endow tunable embolic duration and has a clinically relevant shelf life (Hu et al., 2019).

The current chemoembolization technique dominantly comprises conventional TACE (C-TACE) and drug-eluting bead TACE (D-TACE). C-TACE sequentially delivers a mixture of anticancer drugs and ethiodized oil into the tumor vasculature, normally followed by an embolic particle, whereas for D-TACE the clinicians utilize drug-loadable microspheres, which not only blocks the target vessels but releases the drugs on a controlled manner. Non-calibrated particulates have gradually exited from the bedside apart from a few exceptions (e.g., gelatin pledgets) because the *in vivo* tracking is unpredictable. Therefore, calibrated microspheres are the preferred choice due to their narrow size distribution and defined shape, which helps to improve embolization outcomes (Derdeyn, Moran, Cross, Dietrich, & Dacey, 1995).

The size, distribution, and drug delivery of calibrated microspheres served as key factors to determine the embolization efficiency as well as the procedure’s safety and therapy (Padia et al., 2013; Melchiorre et al., 2018; Boeken et al., 2020). Accurate particle size is crucial for the embolization of tumor-feeding vessels as microspheres are delivered by the force of blood flow and the *in vivo* distribution and accumulation are also size-dependent. For example, in the case of ideal tumor embolization, deep and distal microvascular embolization is often desirable, but too small is also dangerous for vascular embolization as the particles with a mean diameter of 9 μm have been evidenced to cause pulmonary embolic in rabbits due to their high extrahepatic permeability (van Es et al., 1999). The size rule was verified on the VX2 cancer model by scanning the organ distribution of fluorescent microspheres of different sizes after embolotherapy. Overall, 40 μm is the minimum size for particular embolic agents (Bastian, Bartkowski, Kohler, & Kissel, 1998). Typically, microspheres in the range from 40 to 100 μm were suitable for the blockage of the hepatic artery’s end branch. Meanwhile, 300 μm microspheres were more suitable for occlusion of tumor proximal vascular (Lazzaro et al., 2011); while bigger microspheres with a size of (500–900 μm) were regularly applied for uterine artery blockage. Those requirements indicate that the size of embolic microspheres relied on the diameter of the target vessel (Siskin et al., 2000; van Hooy-Corstjens et al., 2008). Therefore, for a size-defined calibrated microsphere it is difficult to fill 100% of the targeted vasculature since the vascular diameter is variable ranging from hundreds of micrometers to several micrometers (7–9 μm for capillary vessel) (Dreher et al., 2012), therefore conventional calibrated microspheres might not be ideal for vascular embolization, and it is urgent to develop more smart, deformable and flexible embolic agents.

To address this dilemma some environment-responsible or smart biopolymers were introduced for vessel embolization. In 2015 Ghandehari et al. developed an *in-situ* gelling silk elastin-like protein polymer for transarterial chemoembolization (Poursaid et al., 2015). Recently we try to introduce an aggregation-responsive particulate embolic agent. In detail, sevelamer ultrafine particle was developed as a highly mobile embolic polymer for chemoembolization therapy, the small particles maximally fill the target artery, as they are ready to aggregate into big particles with the tunable size of hundreds of micrometers by the activation of endogenous inorganic phosphate (Pi), thereby expecting to avoid inter or intrahepatic distribution (Bi et al., 2021). Here we would give evidence of this permeability and challenge the traditional “40 μm” rule by analyzing the specimen after embolization. Furthermore, the therapeutic effects and post-embolization adverse events based on this particular aggregation-embolization will be evaluated by contrast with C-TACE and D-TACE. This Pi-induced embolization strategy not only meets the demand to maximally fill the targeted vasculature but is free from washing out to normal organs.

## 2 Materials and methods

### 2.1 Preparation of sevelamer ultrafine particles and Pi-induced aggregation

Sevelamer hydrochloride (hereafter referred to as sevelamer) was purchased from Wuhan Hezhong Pharmaceutical Co., Ltd., and sevelamer ultrafine particles were fabricated *via* a simple top-down process in a stainless-steel ball. As a microcrystalline polymer, it has an irregular shape with a size of tens to hundreds of micrometers. Sevelamer (2 g in 30 ml DI water) was mixed with 150 g inert ceramic balls (Φ15 mm: Φ5 mm: Φ2 mm = 50 g: 50 g: 50 g) in the autoclave, ground together at the speed of 300 RPM for 24 h by QM-3SP4 planetary ball miller (Nanjing Nanda Instrument Co., Ltd.). After that, the sevelamer was lyophilized and stored at room temperature.

A scanning electron microscope (FEI Quanta 200F) was utilized to record the process of Pi-activated aggregation of sevelamer particles from “nano” to “micro”. In detail, excessive phosphate-buffered solution (PBS) containing a phosphate mass higher than that of the full phosphate binding capacity (5.5 mmol/g) was mixed with sevelamer for 120 min at 37°C. The pristine and Pi-saturated sevelamer were lyophilized for imaging. The hydrated diameter and the surface charge of the ground sevelamer were measured after dispersion in Tris-HCl buffer (pH 7.4) by dynamic light scattering technique (DLS) (Malvern R-Zetasizer Nano ZS90). The measurement was repeated in triplicate.

### 2.2 Protocol of VX2 liver cancer model for embolotherapy

The protocol for VX2 tumor implant and liver tumor generation was described in the reference (Jung et al., 2013; Parvinian, Casadaban, & Gaba, 2014). Briefly, frozen rabbit VX2 tumors were injected into the hind limb muscle of the New Zealand white rabbits for incubation. Around 2–3 weeks after implantation, the donor rabbits were anesthetized with ketamine (22 mg/kg, administered subcutaneously), followed by maintenance with 1%–3% isoflurane, and the hind-limb tumors were excised from the hind-limb and fragmented into several pieces of 1 mm^3^ lesions for liver implantation. Under aseptic conditions, a mini-laparotomy was performed in the subxiphoid area to expose the liver, and one preprocedural dose of enrofloxacin (5 mg/kg, administered subcutaneously) antibiotic prophylaxis was provided to reduce the infection after the surgeries. A tumor fragment was freshly harvested and implanted in the left hepatic lobe of recipient rabbits. A cotton swab was utilized to stop bleeding by pressing the puncture point. The abdomen was then safely closed. After the procedure, the animals were returned to their cages and monitored daily for wound healing and appetite until the TACE procedure. The orthotopically implanted VX2 tumors normally grow up to 1–2 cm^3^ in diameter 14 days later.

A standard embolotherapy on VX2 rabbit was followed. Under the same anesthesia and anti-infective treatment, a surgical cutdown was performed to gain access to the right common femoral artery for vascular access *via* a 4-F vascular sheath (Cook, Bloomington, IN), and then a 4-F catheter (Cordis, Miami Lakes, FL) was inserted into the aorta, subsequently, another 1.98-F catheter (Asahi, Intec, Hanoi, Vietnam) was placed into the celiac axis and the hepatic artery feeding the tumor *via* a 0.018-inch diameter guide wire (Asahi Intec, Hanoi, Vietnam). Digital subtraction angiography (DSA) procedures equipped with DynaCT (Artis Zee Ceiling, SIEMENS) were performed. Different embolic agents in various cohorts were injected carefully. After that, the catheter was removed and the femoral artery was ligated by using resorbable suture material. All the rabbits were returned to cages and daily care until sacrifice or blood sampling at different intervals.

### 2.3 Evaluation of different microspheres’ intrahepatic permeability

Four types of microspheres’ intrahepatic diffusion across the VX2 lesions were evaluated after their transarterial injection as an embolic agent. In the first and second groups, red luminescent polystyrene microsphere (LPM) with the calibrated size of 10 μm (LPM-10) and 100 μm (LPM-100), purchased from Xi’an Rui-xi Biotech, was utilized to compare the permeability across the tumor. In the third group, the commercial poly (vinyl alcohol) microsphere with the size of 100–300 μm (Callispheres^®^, Suzhou Hengrui Biotech) was selected as the standard particulate embolization. In the fourth one, the ultrafine sevelamer microparticles in saline were transarterial administered, and we named it transarterial sevelamer embolization (TASE). Note that in all the groups 2 mg microparticle was equivalently utilized. The whole liver including VX2 liver cancer and adjacent tissues were resected for histopathological analysis 1 h and 24 h after the procedure, respectively.

In the first and second cohorts, we match each slice’s fluorescent and bright field images to evaluate its intrahepatic permeability. In detail, after the autopsy, the whole tissues including liver cancer and adjacent normal tissue were taken out to make frozen sections, to maximally preserve the luminescent polymer within the tissue. Two neighboring slices were selected for matching since the demarcation line between VX2 cancer and the adjacent tissue is not clear from the fluorescence image. Another slice was stained with hematoxylin-eosin (H&E) to figure out the border after being scanned by a digital slice scanner, as the fluorescence and H&E tissue images were matched together it is easy to determine the microsphere’s permeability across the lesion. In the third and fourth group since the commercial microsphere and sevelamer microsphere is inactive luminescence, and according to our experience only H&E pathology is supportive enough to figure out its permeability across the border between the VX2 lesions and adjacent hepatic tissues.

### 2.4 Therapeutic and side effects of different embolic microsphere

In this part all the VX2 rabbit model (*n* = 24) was randomly divided into the following four groups (*n* = 6) with different embolic microsphere: sham group (saline perfusion), D-TACE group (100–300 μm, 0.1 ml Callispheres microspheres (1 g/ml) containing 1 mg doxorubicin), C-TACE group (1 ml lipiodol emulsion with 1 mg of doxorubicin) and TASE group (2 mg sevelamer microspheres). The whole blood was collected from the rabbits’ auricular veins at designed intervals and sacrificed on the 14th day. Harvested tumors and other organs were placed into 10% neutral buffered formalin.

Measurement of tumor necrosis. —Tumor necrosis was identified as the primary response outcome measured herein, given that tumor necrosis is normally related to survival after local regional therapy for HCC (Holmes-Farley, Mandeville, Ward, & Miller, 1999; Jung et al., 2013). Tumor necrosis was expressed as a percentage of tumor area for each section (%necrosis in the following equation). To quantitatively estimated necrosis, visual inspection with the accurate region of interest generation around the whole tumor and necrotic portions was employed by calculated as follows: Necrosis% = necrotic area necrosis/whole tumor area×100. The percentage of tumor necrosis across three analyzed tumor sections automatically read by the software “image-pro plus 6.0” was averaged to provide a tumor necrosis fraction for each tumor.

Toxicity analysis of serum biochemistry—2 ml blood was taken from the ear vein of rabbits 1 day before and 3-, 7-, and 14 days post-operation. After centrifugation at 3500 r/min for 10 min, the supernatant was taken out for quantitative measurement of main liver and kidney function parameters including aspartate aminotransferase (AST), alanine aminotransferase (ALT), serum urea nitrogen (BUN) and creatinine (CREA) by select-2 automatic biochemical analyzer.

Toxicity analysis of histopathology—Tissue specimens including the normal liver, heart, and lung was preserved after the autopsy, and finally, the specimens were made into pathological sections. Hematoxylin and Eosin staining of tumor slices in all four groups were evaluated at up to ×400 magnification for systemic toxicity (apoptosis, necrosis, inflammation, fibrosis). The slides were evaluated in a blinded fashion by a toxicologist (Yan-shu Li, with more than 10 years of experience).

### 2.5 Statistical analysis

Image-Pro Plus software (version 6.0) was utilized for quantitative image analysis. Statistical analyses were performed by GraphPad Prism software (version 8.0), and Origin Pro software (version 9.0) for the DLS curve. Comparisons between groups were calculated by Student’s t-test and one-way ANOVA. The level of statistical significance was set at *p* < 0.05.

## 3 Results

**FIGURE 1:**
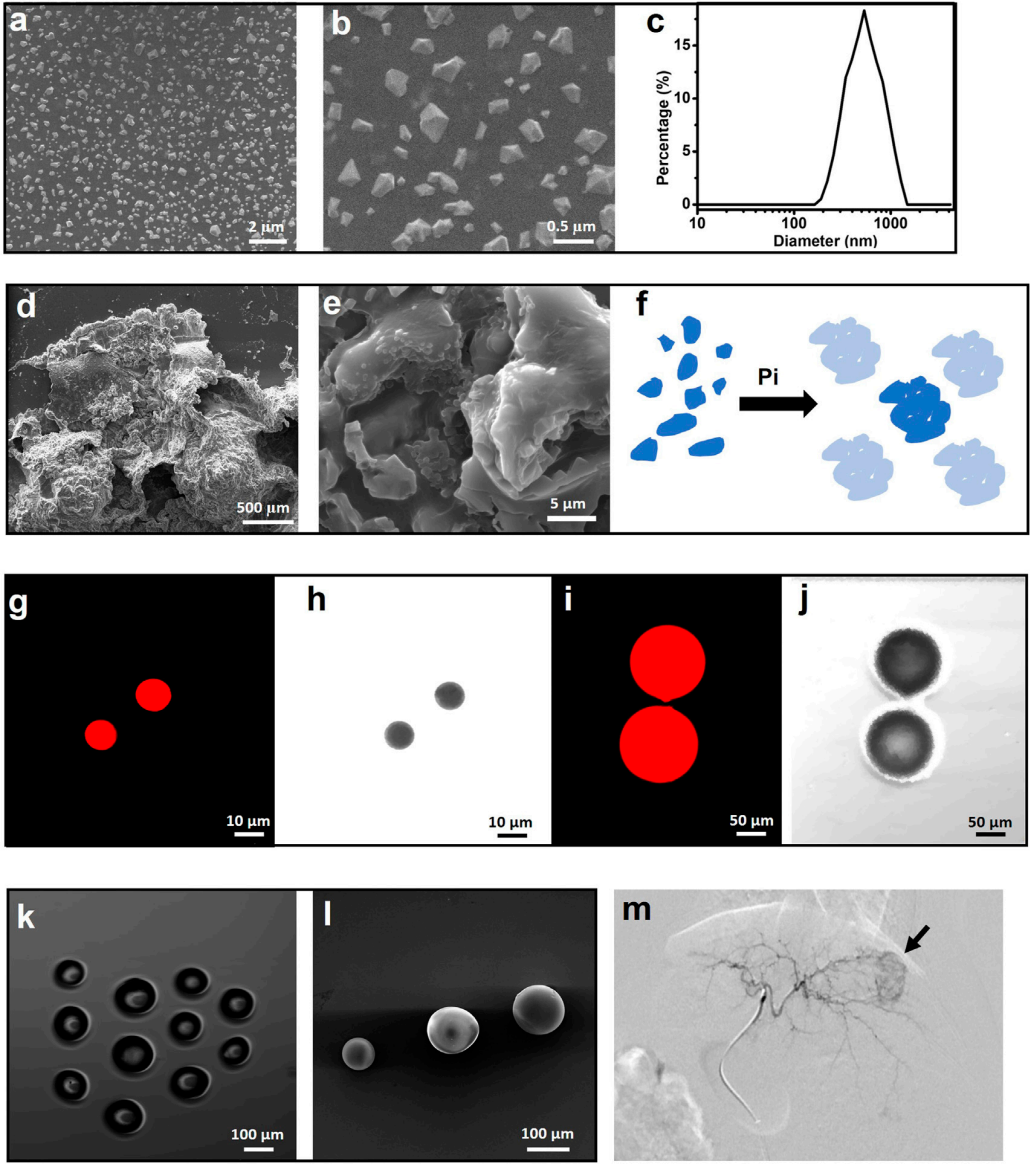
Polyhedral sevelamer particle with a size ranging from 0.2–0.5 µm **(A–C)** and it is Pi-induced swell and aggregation into particles as large as millimeter **(D,E)**, as simply schemed **(F)**. Luminescent polystyrene microsphere (LPM) with the size of 10 μm **(G,H)** and 100 μm **(I,J)**, as recorded by a luminescent microscope. Light microscope **(K)** and scanning electron microscope **(L)** images of poly (vinyl alcohol) microsphere with the size of 100–300 μm. **(M)** Representative VX2 rabbit hepatic angiographic images.

Sevelamer hydrochloride (simply named sevelamer, 1,016 Da) is a cationic phosphate-binding agent. It is hydrophilic but insoluble in water. Sevelamer rapidly absorbs phosphate anions through ion exchange and hydrogen bonds and forms insoluble, non-absorbable sevelamer-phosphate complexes. This absorption is highly effective with a phosphate binding capacity of 5.5 mmol g^−1^ in a wide optimal pH range from 4.5 to 7.5 (Holmes-Farley et al., 1999). Commercial sevelamer is normally crystallized with the size of hundreds of micrometers (Swanson et al., 2013). After being ground for hours, it was fragmented into polyhedron particles with a size ranging from 0.2–0.5 μm (Figures 1A, B). DLS revealed that the hydrated diameter is 0.689 ± 0.365 μm with a polydispersity index (PDI) of 0.48 and a positive charge of 20.28 mV. The morphology of the sevelamer before and after absorption of Pi remarkably varied from the individual ultrafine particle to hard aggregates (Figures 1C, D) as phosphate ions induced adjacent crystal face swelling and connection (Figure 1E), and the whole process of Pi induced “nano” to “micro” aggregation was schemed (Figure 1F). The red luminescent polystyrene microsphere (Figures 1G–J) with a calibrated size of 10 and 100 μm was utilized to verify the so-called “40 μm” rule. Commercial embolic bead without loading drugs (Figure 1K, L) has a defined size ranging from 100 to 300 μm. Microsphere-based embolization involves the target delivery of the four types of particles directly into a tumor’s blood-feeding vessels under the guidance of DSA, as normally shown in figure 1 m.

### 3.1 Microsphere’s permeability

**FIGURE 2:**
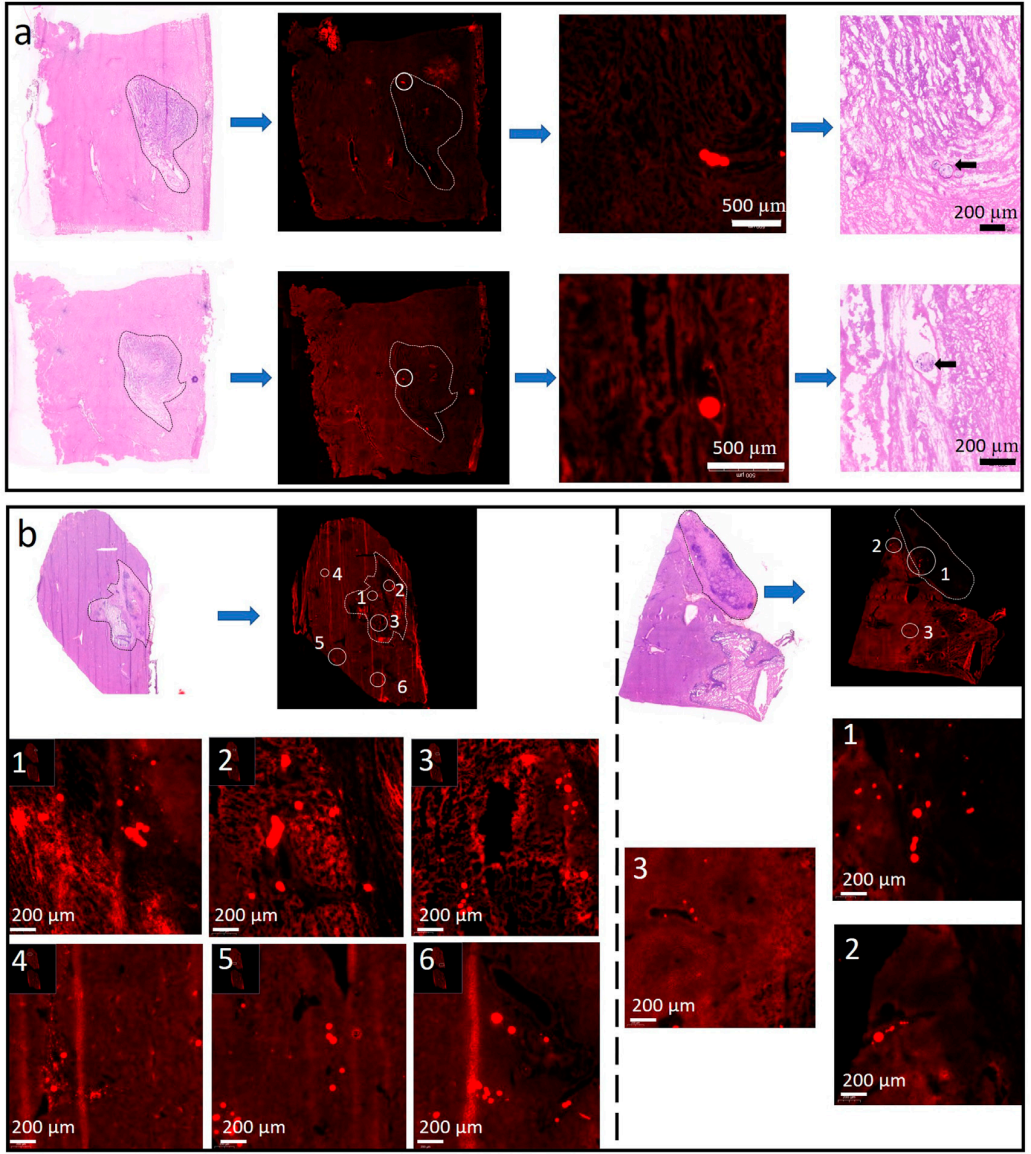
Representative slices are indicative of LMP-100 **(A)** and LMP-10 **(B)** distribution 1 and 24 h after the procedure. Note that all the luminescent images were made by frozen sections on the neighbor slice of the H&E-stained section to position and discriminate the MPs.

The “40 μm” rule on the VX2 carcinoma model was verified by microscopic analysis of both LPM’s distribution in the Rabbit’s liver lobe and hepatic VX2 tumor. The slide prepared by the standard frozen sections was compared with the neighboring slide made by H&E staining protocol, and then both are examined by fluorescence microscope and optical microscopy scanner, respectively. As shown in Figures 2A, B, H&E staining was utilized to define the tumor boundary as the white discontinuous line figured. All the single MP, as well as their agglomerations, emits red light on all the slides. Figure 2A the LPM-100 intra-and extra-tumoral distribution 1 (above) and 12 (bottom) hours after the procedure, and it is only detected inside the tumor, dominantly the inner edge of the tumor due to possible fluid pressure between the intratumor and extra tumor. Note that the red luminescent spot outside of the tumor does not come from LPM-100 after magnification. Comparably, LPM-10 was found both in the tumor and the adjacent liver lobe, whether in the initial period or late stage (Figure 2B) and normally, in the early stage (1 h) more LPM-10 individual or agglomerates (left part) was visible inside VX2 tumor. As time goes by (24 h), it is rare to find LPM-10 inside the tumor, and most of them were accumulated outside the tumor (right part). The LPM’s permeability is consistent with the reported phenomena (Pillai et al., 1991), that is, smaller MP than 40 µm are easy to be washed out from the liver tumor, and transported to intrahepatic or extrahepatic tissue. The MP larger than 40 µm was mainly trapped directly within the tumor. This guarantees a complete embolization with the temporary or permanent arrest of the blood flow, depending on its bio-stability.

**FIGURE 3:**
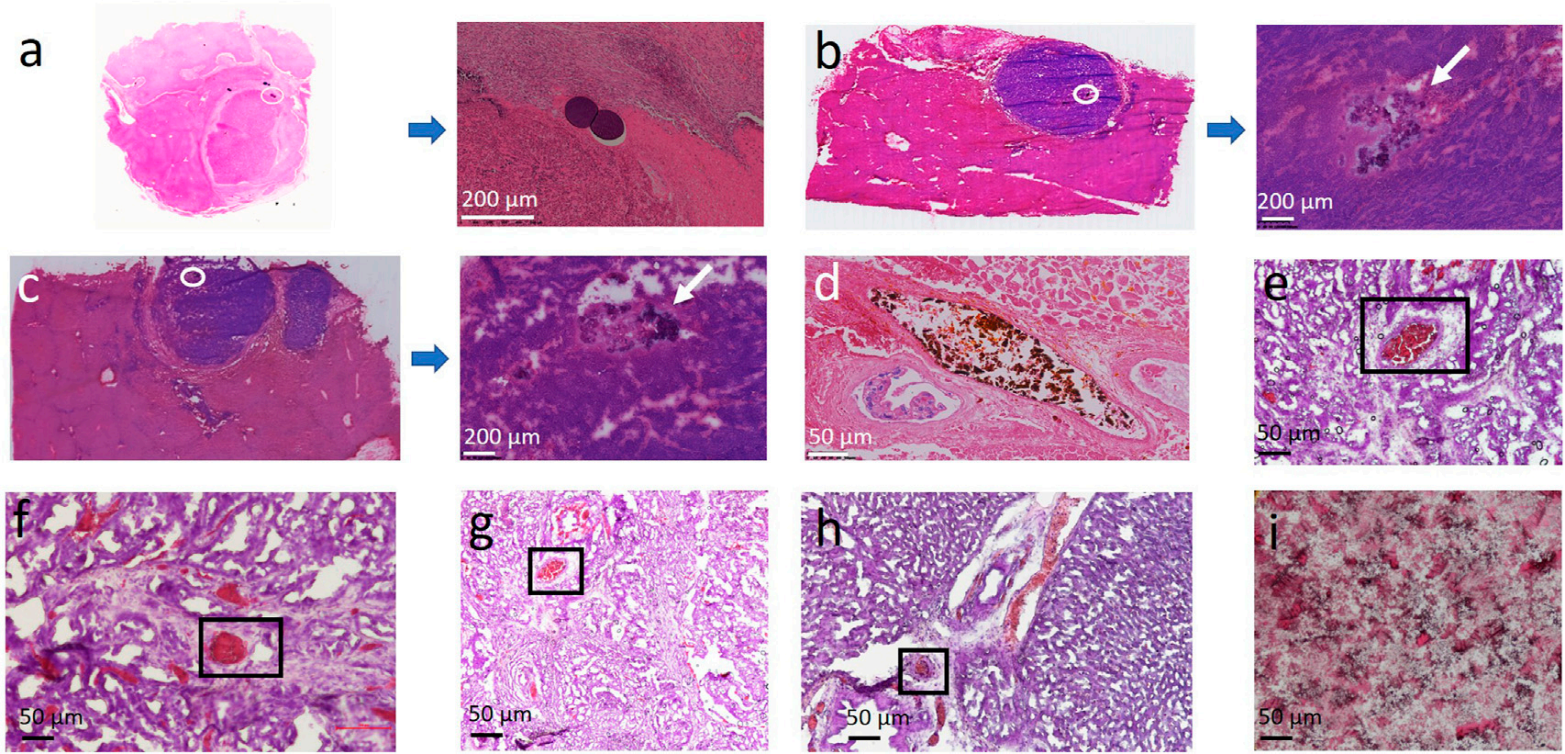
**(A)** Representative slices of commercial bead trapped VX2 carcinoma. **(B,C)** Representative slices of sevelamer trapped VX2 carcinoma and their local magnifications 1 and 24 h after the procedure. **(D,E)** Some sevelamer particles were aggregated within the big **(D)** or small vessels **(E)** due to their deformability. The vessels with a size around or below 40 µm are also found to be fully embolized by the aggregates **(F–H)**. Hematoxylin-eosin staining of pure sevelamer was set as control **(I)**.

As expected, commercial PVA bead (100–300 µm) is exclusively observed within the scope of VX2 carcinoma, 24 h after embolotherapy (Figure 3A). To be mentioned, the MP density might be not as high as expected due to the repeated washing or staining during the preparation of the slice by the conventional H&E staining process. The permeability of sevelamer particles is abnormal, opposite to the conventional “40 µm”. Although its is size only around 1 μm, at both time points (1 h in Figure 3B, 24 h in Figure 3C) we have not observed it overflow from the VX2 carcinoma into the adjacent liver lobe, as LPM-10 did. On the contrary, sevelamer particles swelled and united into big aggregates, as the white arrow points out in Figures 3B, C. Sometimes we also found the aggregates were trapped within the vessels with different diameters 24 h later, indicating that due to its shape deformability it could occlude big as well as small vessels (Figures 3D–H), which might assure the complete vessel embolization. Note that there is no chance to find the sevelamer MP outside of the VX2 carcinoma.

### 3.2 Tumor necrosis

**FIGURE 4:**
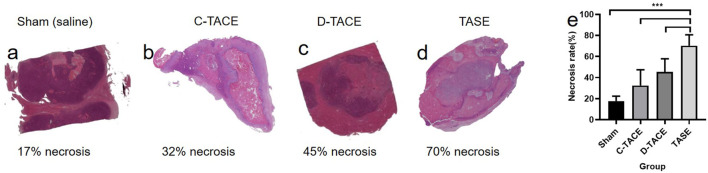
**(A–D)** Photomicrographs (hematoxylin-eosin stain; original magnification) 1 week after sham (saline), C-TACE, D-TACE, and TASE treatment is indicative of demonstrating increasing tumor necrosis. **(E)** the statistical results of necrosis rates in the four groups (*n* = 6). NS = *p* > 0.05, *p < 0.05, **p < 0.01, ***p < 0.001, and ****p < 0.0001.

Tumor necrosis was different in the groups (Figure 4). Subset analysis indicated that the increase of tumor necrosis treated by TASE (70.17 ± 10.42) was highly significant compared with the sham group (17.67 ± 4.63, *p* < 0.001), moderately significant compared with the C-TACE (32.33 ± 15.08, *p* < 0.001), as well as D-TACE group (45.33 ± 12.50, *p* < 0.001). The comparison indicates that, as a new embolic particulate, sevelamer has a better therapeutic effect than commercial TACEs. D-TACE can load more doses of the drug than 1 mg, even sometimes tens of milligrams. Here TASE is, strictly speaking, a transarterial embolization (TAE) technique without adding an extra anticancer drug. However, clinically TACE is more common than TAE, so here we compare TASE with both clinical methods, instead of focusing on the dose issue.

**FIGURE 5:**
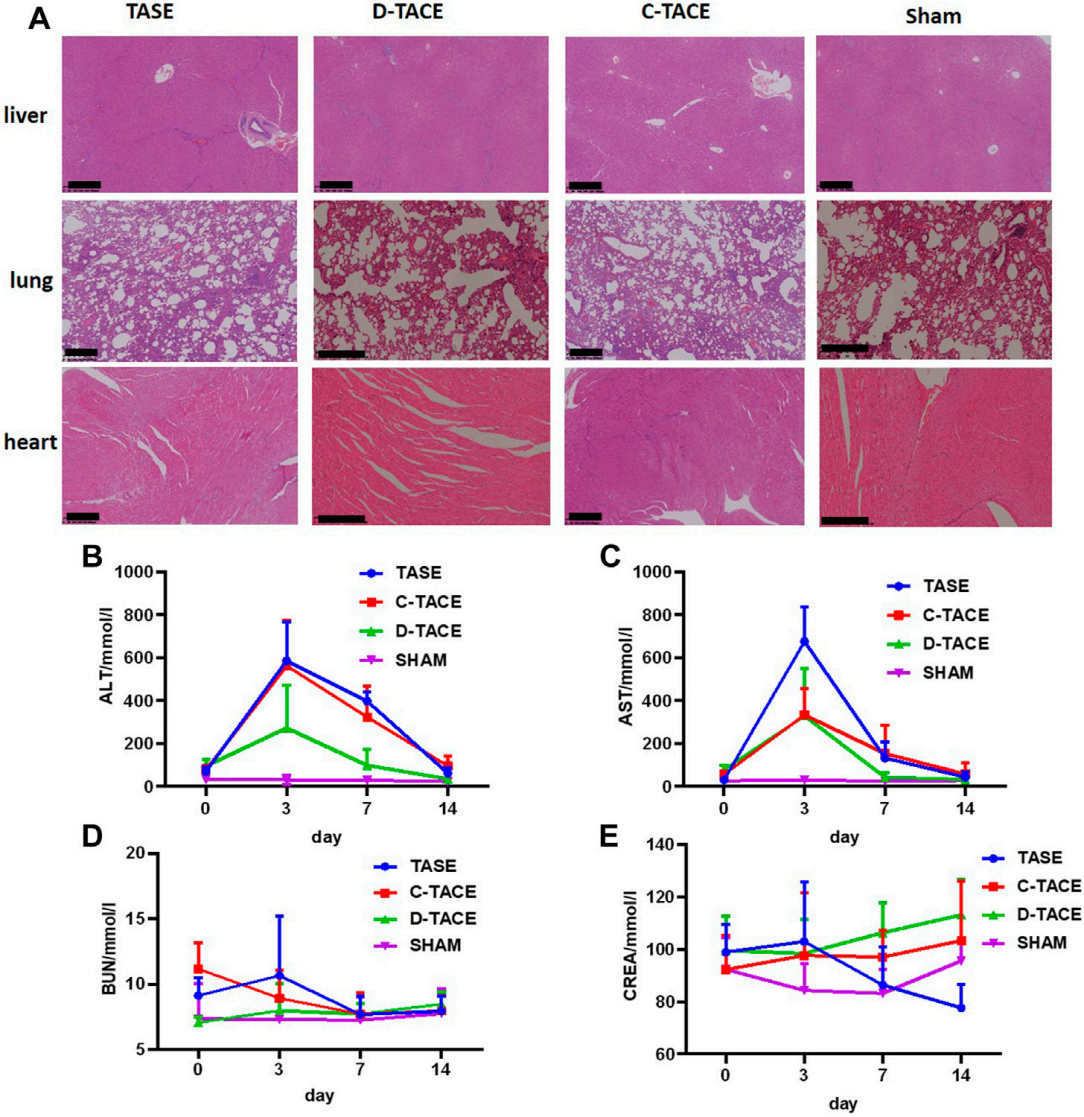
**(A)** H&E staining of organ sections to evaluate the safety of TASE. H&E-stained tissue sections of major organs including liver, lung, and heart of the VX2 rabbit 14 days post-administration. Saline-treated rabbits were used as the control (Scale bar: 500 μm). Blood biochemistry and complete blood analysis of the VX2 rabbit in the four groups at the various time points (the third, seventh, and 10th-day post-procedure). The examined parameters included **(B)** alanine aminotransferase (ALT), **(C)** aspartate aminotransferase (AST), **(D)** serum urea nitrogen (BUN), and **(E)** creatinine (CREA).

The potential *in vivo* toxicity of all the particulates was then systematically evaluated by blood biochemistry and organ histological examination after the procedure. As revealed by histological examination, no obvious histopathological abnormality or lesion was observed in the main organs in all the groups (Figure 5A). Furthermore, all the measured blood biochemistry parameters in the embolization-treated rabbits appeared to be normal at all the time points when compared to those of the sham group (Figure 5B). In conclusion, no appreciable systemic toxicity *in vivo* induced by sevelamer was observed at the designed dose within 14 days.

## 4 Discussions

Particulates were the first embolic agents to be developed and are currently the most commonly used agent due to their versatile functionality (Sheth et al., 2017). Lipoid-based c-TACE remains the most well-applied transarterial therapy for HCC, and clinicians have a growing interest in liquid/gel embolic agents for peripheral interventions because they can penetrate targets that catheters and coils cannot reach. Lipiodol in the chemical formulation of ethyl esters conjugated with iodinated fatty acids extracted from the natural poppyseed oil is extensively used as the liquid radiopaque contrast agent. It has a proper viscosity, slightly denser than water. Lipiodol penetrates the capillary due to its fast diffusivity, thereby it may serve as a capillary embolic agent to obtain capillary stasis (Konya, Stephens, & Wright, 2010) (Becker, Preul, Bichard, Kipke, & McDougall, 2007). However, due to tissue clearance and blood scouring, Lipiodol is easily to be rapidly eliminated, thereby sometimes vascular recanalization can be observed, reducing its embolic efficacy. Other adverse events including pulmonary and cerebral embolism, hypersensitivity reactions, and exacerbation of chronic liver disease must be considered. Additionally, another disadvantage of liquid embolic agents is that low viscosity is preferable to pass through the delivery system (e.g., syringe and catheter), while the formation of a stiffer structure is desired after exiting the catheter if the embolic agent behaves like a cement the vessel casting will be achieved.

Fortunately, polymers could deform *via* phase transition from liquid-to solid-like (sol-gel), and the approaches are normally categorized into chemical crosslinking (e.g., polymerization) and physical crosslinking (e.g., precipitation and ionic crosslinking) (Becker et al., 2007; Brennecka, Preul, Becker, & Vernon, 2013). In particular, the various triggering factors in the tumor physicochemical environment, such as pH, ionic strength, and temperature, altogether provide the driving force to induce crosslink (Zhao et al., 2011). The era of the microsphere is coming. Several kinds of embolic microsphere are on the way to the bedside or have been approved. The size of the microsphere is the biggest issue up to date, and 40 μm is an acceptable lower limit for particulate embolic agents, according to the criteria of an ideal embolic agent. It seems that it cannot overcome the disadvantage due to the size limitation (Lubarsky, Ray, & Funaki, 2009; Lubarsky, Ray, & Funaki, 2010).

Herein we illustrated the third approach: endogenous Pi induced *in situ* aggregations of sevelamer microparticles for artery occlusion. Sevelamer has at least two advantages for artery embolization, firstly, this insoluble polymer is easy to be fragmented into nanoparticles, and the ultrafine nanoparticles are highly mobile and behave like a liquid or gelation agent; secondly, its aggregation is responsive to endogenous Pi ions, during this process Pi inserts into the framework of sevelamer, inducing it to swell and aggregate into bigger particulate, and thereby occluding the tumor-feeding vessels. Due to its small size, sevelamer will go through the whole blood vessel on the proximal end and penetrate deep blood capillary. Eventually, Pi-absorption induces sevelamer aggregation and full embolization after arterially administered (Q. C. Bi et al., 2022).

## 5 Conclusions

In conclusion, we present an illustration of a Pi-induced deformable microparticle for vascular embolization, which might meet the criteria of an ideal embolic agent. In another way, since sevelamer is a commercial oral Pi binding agent, this work also helps to expand the application of sevelamer.

## Data Availability

The original contributions presented in the study are included in the article/supplementary material, further inquiries can be directed to the corresponding author.
